# Patient and health professional attitudes towards the use of telemedicine for abortion care in Britain: Findings from the SACHA study

**DOI:** 10.1177/20552076241288717

**Published:** 2024-11-03

**Authors:** Rebecca Meiksin, Maria Lewandowska, Rachel H Scott, Melissa Palmer, Ona McCarthy, Natasha Salaria, Patricia A Lohr, Jill Shawe, Rebecca Sophia French, Kaye Wellings, the SACHA Study Team

**Affiliations:** 1 Faculty of Public Health and Policy, 4906 London School of Hygiene and Tropical Medicine, London, UK; 2 Faculty of Epidemiology and Population Health, 156606 London School of Hygiene & Tropical Medicine, London, UK; 3 Centre for Reproductive Research & Communication, 146910 British Pregnancy Advisory Service, Stratford-upon-Avon, UK; 4 Faculty of Health, School of Nursing and Midwifery, 6633 University of Plymouth, Plymouth, UK

**Keywords:** Telemedicine, digital health, abortion

## Abstract

**Introduction:**

Use of telemedicine in abortion care is safe and effective. Patient satisfaction with telemedically supported abortion is high, but as use expands in Britain, little is known about patients’ or health professionals’ views on how it is best used. We sought the views of both groups on telemedicine's role in abortion provision and how its use might be optimised.

**Methods:**

We administered a structured survey with an additional free-text response option to a range of health professionals from services identified via clustered random sampling. We conducted semi-structured interviews with patients with recent experience of abortion, purposively sampled from abortion services. Participants were recruited from England, Scotland and Wales. We analysed qualitative data using thematic analysis alongside framework analysis.

**Results:**

Support for telemedicine was high among participants, which included 771 health professionals from a range of services and 48 patients. Among health professionals, 23% opposed the use of telemedicine in abortion provision. Opposition was highest among those uninvolved, or who did not feel skilled, in remote provision. Reported benefits included patient convenience and comfort. Patient concerns centred on adequate provision of emotional support. Participants made suggestions for augmenting and extending the use of telemedicine to meet some patients’ needs, including combining telemedical consultation with in-person care and providing information and follow-up via digital methods.

**Conclusions:**

Acceptability of telemedically supported abortion, which offers logistical and emotional benefits to patients, is high in Britain. Novel ways of providing telemedical support should be explored to enhance remote abortion provision. In-person care should remain an option for patients.

## Introduction

Telemedicine, the adoption of remote health care using telecommunication methods such as telephone, video calls and other Internet-based communication,^1^ is increasingly used in abortion provision.^2^ Evidence shows that telemedically supported medication abortion (MA) is as safe and effective as provision in clinics^3–5^ and more cost-effective.^6,7^ This model of care increases early access to abortion^3,8^ and is acceptable to patients and health professionals,^3,5,9,10^ serving to enhance patient comfort, convenience and privacy^10–12^ and to reduce the stigma.^8,10^ Telemedicine is recommended as an option to improve patient access to abortion by the Royal College of Obstetricians and Gynaecologists and the National Institute for Health and Care Excellence (NICE).^1,13^

As in several other countries, the use of telemedicine in Britain accelerated during the COVID-19 pandemic^14^ to reduce viral transmission.

Medical abortion involves the use of mifepristone followed 1–2 days later by misoprostol. Prior to 2018, by law both medications had to be administered in a clinic, but in almost all cases, the patient went home after their use and passed the pregnancy at home. A remote, 24/7 telephone line was routinely made available to patients by service providers for aftercare. Approval for home use of misoprostol was granted in England, Scotland and Wales in 2018. At the start of the pandemic, approval was also granted for the home use of mifepristone.^15^ This change made possible entirely remote provision of MA which involves a video or phone consultation and the posting of medication to the patient's home or collection from a clinic followed by use at home. Legislation has since permanently extended the approval for home use of both mifepristone and misoprostol,^16^ embedding telemedicine as a routine component of abortion care in Britain. Supported by telemedical care, MAs rose as a proportion of all abortions in England and Wales from approximately 70% in 2019 to 87% in 2021^14^ and in Scotland from 88% to 99%^17^ during the same period. Self-administering both mifepristone and misoprostol at home is now the most common abortion procedure in England and Wales, accounting for 61% of abortions in England and Wales in 2022.^18^

Satisfaction with telemedical abortion care has been shown to be high; between two-thirds and three-quarters (66–74%) of patients surveyed in Britain would choose MA via telemedicine should the need for abortion recur.^9,10^ Little is known about the possible reservations of the minority who would not choose this model of care again.

Studies describe a range of telemedical models of abortion care, from phone or video consultations as a complement to in-person care to entirely remote abortion provision, as described above.^5^ Telemedicine can be used at different points in the patient journey, from decision-making and referral/access through to the abortion procedure and aftercare. Little is known about factors that affect patients’ preferences at different stages of the abortion process, which patients are most likely to find telemedical provision helpful, how its use can be optimised and how and when telemedicine might best augment or be complemented by in-person care. Calls have been made for greater attention to users’ perspectives on telemedical approaches.^19^ Furthermore, little is known in Britain about the views of health professionals towards remote provision who are or could be involved in providing this or about their level of confidence in doing so. To address these gaps, we recruited patients with recent experience of abortion, as well as health professionals from a range of services, across Britain. We asked their views on the role of telemedicine in abortion care and support. We aimed to address the following research questions:

1. How acceptable do health professionals and patients find telemedicine for abortion?
2. How well do perspectives of health professionals and patients align on the role of telemedicine in abortion?
3. Where do telemedical approaches need to be complemented by in-person care, and where might the role of telemedicine be usefully extended?

We carried out this work out as part of the Shaping Abortion for Change (SACHA) study,^20^ a large, multi-component study which aimed to provide a broad evidence base to inform future directions for the regulation, governance and organisation of abortion provision in Britain. Previous peer-reviewed publications drawing on the SACHA study findings have reported on a review of interventions and new models of care for medical abortion at home^11^; patients’ perspectives on how patient experience might be improved^21^ and on arrangements for abortion provision during the COVID-19 pandemic^12^; and patient and public involvement in the study.^22^ The present paper reports patients’ and health professionals’ perspectives on the use of telemedicine for abortion care and support and on how its use might be optimised.

## Methods

### Survey of health professionals

#### Recruitment, eligibility and data collection

We distributed a survey for completion by a range of health professionals exploring their attitudes, knowledge and practices regarding abortion provision. Using clustered random sampling in England, Scotland and Wales, stratified by country, 278 sites were selected and invited to take part from general practice, sexual and reproductive health (SRH) clinics, specialist abortion services, maternity services and pharmacies. All doctors, nurses, midwives and pharmacists working in the selected sites (and within a selected 24-hour period, for maternity services) were eligible to take part, including both current and potential providers of abortion care. These staff were identified, and questionnaires were distributed between November 2021 and July 2022. The survey comprised a structured and fully scheduled questionnaire, developed by the study team and piloted with representatives from professional groups and services of interest, and it included space for additional free-text comments.

We sent health professionals packs containing a paper questionnaire with their unique identification number, a prepaid return envelope, a participant information sheet and an unconditional £10 thank-you voucher. The pack also included instructions for completing the survey online if preferred. We followed up the initial mailout with two emails or phone calls to non-responders. Returning the self-completed questionnaire or submitting it online implied consent. The need for separate written consent was waived by the four ethics committees providing ethical approval for the health professionals’ survey (please see ‘Ethical approval’ section below for the details of each committee). Following data entry, we exported quantitative data into Stata 17^23^ and free-text data into Excel,^24^ for cleaning and analysis.

Further details of sampling, recruitment and data collection methods for the survey are provided elsewhere.^25^

#### Measures

Attitudinal statements in the provider survey presented a mix of viewpoints to reduce acquiescence bias. The outcome variable in this analysis was health professionals’ view of the acceptability of using telemedical support in abortion provision, operationalised by response to the statement, ‘Digital technologies, e.g., via video, are not an acceptable way to provide abortion care/support’. Independent variables were personal characteristics of health professionals (gender, age, country, importance of religion, political beliefs), professional characteristics (time since qualification, service type, profession), current involvement in abortion provision and views on the appropriate setting for abortion provision.

#### Analysis

We estimated the proportion (with 95% confidence interval [CI]) of respondents agreeing with the statement on the acceptability of digital technologies overall and by each independent variable, accounting for clustering by health service. We analysed free-text comments relating to the use of telemedicine in abortion provision using the framework approach.^26^ We subjected free-text data categorised as relating to ‘telemedicine and self-management’ to inductive thematic analysis and then organised these data in a spreadsheet using Microsoft Excel^24^ according to the emergent themes ‘overall attitudes towards telemedicine’, ‘standard of care’, ‘access’ and ‘need for ultrasound’. We summarised findings within stages of the patient journey where appropriate.

### Interviews with patients

#### Recruitment, eligibility and data collection

We purposively sampled patients from a range of abortion services. Criteria for eligibility were as follows: aged 16 years and older; living in Britain; able to take part in an interview in Arabic, English, Polish or Welsh; receiving abortion care for reasons other than foetal anomaly at a participating independent or NHS site in England, Scotland or Wales; and able to provide informed consent. Study flyers were placed in clinical units of participating sites, and eligible patients were informed about the study during booking or their initial consultation. If interested in learning more, they were able to meet with a researcher on-site or to contact or be contacted by a member of the study team later via their preferred method (telephone, SMS and/or email). As the study progressed, the research team monitored participant characteristics and tailored subsequent recruitment to achieve diversity in the sample in relation to age, ethnicity and method of abortion.

Trained researchers conducted semi-structured, in-depth interviews by telephone or video between July 2021 and August 2022 with patients 2–8 weeks post-abortion. All patients received a patient information sheet. Patients provided written (for in-person interviews) or audio-recorded oral informed consent prior to their interview. We asked about patients’ experience of and views on abortion provision including the use of telemedicine throughout the patient journey. We also asked about their age; ethnic group; relationship status; involvement in work, education and/or training; living arrangements; and previous pregnancies. Interviews were audio-recorded and transcribed verbatim. We offered patients £20 vouchers in appreciation of their time.

### Analysis

We imported interview transcripts into NVivo 12^27^ and analysed these using thematic analysis in conjunction with the framework approach.^26^ Following familiarisation with the data, we applied pre-established codes relating to stages of the patient journey (decision-making, referral and access, abortion procedure and aftercare) and applied open coding to data within each stage to inductively identify emerging themes relating to patient attitudes; perceived drawbacks and benefits; and recommendations. We paid close attention to deviant cases, which we report alongside dominant themes.^26^

### Results

Of 1370 questionnaires distributed to eligible health professionals, 771 (56.3%) were returned. Characteristics of the achieved health professional sample have been reported elsewhere and are shown in Appendix A.^25^ Of the total sample, 21 respondents – all but one of whom were currently involved in abortion care – provided free-text comments relating to use of telemedicine. Forty-eight patients aged 16–43 years with recent experience of abortion took part in in-depth interviews. Patient characteristics have been reported elsewhere^21^ and are shown in Appendix B.

### Survey responses: health professionals’ attitudes towards telemedicine

Of all health professionals responding to the survey, 46.9% (95% CI 39.4–54.6) disagreed with the statement ‘Digital technologies, e.g., via video, are not an acceptable way to provide abortion care/support’, 22.9% (95% CI 18.3–28.4) agreed, and 30.2% (95% CI 25.6–35.3) neither agreed nor disagreed (Table 1). To aid clarity, we describe agreement with this statement as opposition to digital technologies in abortion provision. In terms of personal characteristics, participants’ views varied by political stance and the importance of religion in their life, with opposition to digital technologies being highest among those with political views that were right or right of centre (36.7%, 95% CI 22.2–54.0) and among those for whom religion was very important (40.0%, 95% CI 27.6–53.9). A greater percentage of participants in England (25.6%, 95% CI 20.4–31.6) than those in Scotland (9.4%, 95% CI 4.9–17.3) was opposed to digital technologies in abortion provision.

**Table 1.** Health professionals’ attitudes towards the use of telemedicine in abortion provision.

Health professionals’ characteristics	Agreement with statement: ‘*Digital technologies, e.g. via video, are not an acceptable way to provide abortion care/support*’		
	**N**	**%**	**95% CI**
**Total**	(N = 762)		
Agree	174	22.86	[18.32,28.14]
Neither agree nor disagree	230	30.22	[25.59,35.29]
Disagree	358	46.91	[39.36,54.61]
**Gender**	(N = 759)		
Female	156	23.49	[18.53,29.30]
Male	18	19.57	[12.67,28.97]
**Age group**	(N = 760)		
Under 30	17	20.24	[13.47,29.26]
30–39	50	24.15	[18.50,30.88]
40–49	41	19.71	[13.81,27.33]
50 and over	66	25.38	[18.87,33.22]
**Years since qualification**	(N = 758)		
< 5	21	21.65	[14.51,31.03]
5–10	38	22.89	[17.07,29.98]
11–20	51	23.94	[17.89,31.27]
>20	64	22.78	[16.96,29.87]
**Country**	(N = 761)		
England	142	25.59	[20.41,31.55]
Scotland	12	9.38	[4.88,17.25]
Wales	20	25.64	[16.02,38.40]
**Importance of religion**	(N = 759)		
Very important	24	40.00	[27.57,53.87]
Quite important	33	22.76	[15.59,31.98]
Not important	104	20.12	[15.61,25.53]
**Political beliefs**	(N = 756)		
Right/right of centre	11	36.67	[22.20,54.02]
Centre	28	25.00	[18.09,33.47]
Left/left of centre	32	14.16	[9.09,21.38]
None	76	25.17	[19.02,32.49]
**Service type**	(N = 761)		
Specialist abortion	26	10.66	[6.53,16.92]
General practice	37	24.03	[18.12,31.13]
Maternity	73	37.06	[28.68,46.29]
Pharmacy	19	36.54	[25.42,49.30]
SRH clinic	19	16.67	[11.97,22.73]
**Profession**	(N = 756)		
Doctor	22	12.72	[8.35,18.90]
Midwife	79	30.15	[21.67,40.24]
Nurse	49	18.85	[14.21,24.56]
Pharmacist	22	36.67	[26.49,48.19]
**Clinic/hospital should be attended for abortion**	(N = 760)		
Agree	79	52.32	[44.06,60.45]
Neither agree nor disagree	39	22.94	[16.15,31.52]
Disagree	55	12.56	[8.80,17.61]
**Feels adequately skilled to support home management**	(N = 760)		
Yes	37	13.17	[8.54, 19.76]
No	136	28.45	[23.72,33.71]
**Currently providing abortion**	(N = 754)		
Yes, on-site only	40	28.78	[21.11,37.89]
Yes, any remote	41	12.69	[8.97,17.67]
No	90	30.93	[25.73,36.66]

Note: Denominators exclude missing values.

In terms of the service type, health professionals working in SRH care services and specialist abortion providers were least opposed to the use of digital technologies for abortion care, with only one in six (16.7%, 95% CI 12.0–22.7) and one in ten (10.7%, 95% CI 6.5–16.9), respectively, considering it to be unacceptable. Opposition to use of digital technologies in abortion care was lower among doctors (12.7%, 95% CI 8.4–18.9) than among midwives and pharmacists and higher among pharmacists (36.7%, 95% CI 26.5–48.2) than among doctors and nurses. It was lower among health professionals who did not see the need for women to attend a clinic/hospital to have an abortion (12.6%, 95% CI 8.8–17.6) compared with those who did (52%, 95% CI 44.1–60.5). Opposition to the use of digital technologies in abortion care was also lower among those who felt skilled in supporting women in abortion home management (13.2%, 95% CI 8.5–19.8) compared with those who did not feel skilled (28.5%, 23.7–33.7) and among those currently involved in providing abortion care remotely (12.7%, 95% CI 8.97–17.7) compared with those involved on-site only (28.8%, 95% CI 21.1–37.9) or not at all (30.9%, 95% CI 25.7–36.7).

### Free-text responses from health professionals

Of the 21 survey respondents providing free-text responses about telemedicine, 13 expressed concerns about telemedicine and 8 identified benefits.

### Perceived benefits of telemedical abortion care

Comments in support of telemedicine highlighted increased access – especially among patients experiencing domestic abuse, greater comfort and convenience for patients and savings in time or money. Perceived benefits also included a lessening of anxiety and greater ease in discussing personal details. Benefits to the health service were also mentioned, including cost savings and, as reported by this nurse working in a dedicated abortion service, increased capacity to see more patients: ‘[Telemedicine] has improved access to abortion services. Our service is extremely busy and we would not cope if we had to return to every patient /woman having a face-to-face consultation’ (HP14, nurse, abortion provider in dedicated abortion service, Scotland). A nurse involved in abortion care in England commented, ‘Risk is with everything but if benefit[s] outweigh the risk it is worth it’ (HP10, nurse, abortion provider in dedicated abortion service, England).

Survey evidence that opposition to telemedical abortion provision was lower among health professionals with experience of abortion provision was amplified by free-text comments. Initial concerns about telemedical care were allayed by familiarity with its use for one abortion provider:

For me, the service has required a change in working conditions, using telephone/ video for consultations, I was concerned, initially, at this lack of face to face with clients, but after working remotely for over a year now, I get the same job satisfaction as I always did. (HP21, midwife, abortion provider in dedicated abortion service, England)

### Perceived limitations of telemedical abortion care

Free-text comments from some healthcare professionals revealed a perception that a phone consultation may limit the capacity of providers to give comprehensive advice and information to some patients. The main concern raised by health professionals, however, focused on the risks they thought may be attendant on MAs taking place without having had a pre-treatment ultrasound. These included the potential medical consequences of delayed or undiagnosed ectopic or molar pregnancies or where the gestational age was more advanced than had been thought. As one abortion provider wrote:

I do believe they should have an ultrasound prior to a TOP [termination of pregnancy]. We completed these during the COVID lockdown. We have had young girls believe they are 6 weeks, and one was 23 weeks. W[e] have also had ectopic pregnancies and molar pregnancies. (HP18, nurse, abortion provider in dedicated abortion service, England)

Some participants described rare emergencies that had occurred after MA, including an instance of the need for emergency gynaecological care due to bleeding for a patient whose pregnancy was more advanced than previously thought. One comment recommended stricter assessment criteria to identify suitable candidates for self-managed abortion without a pre-treatment ultrasound and to identify patients who should attend for an in-person ultrasound.

### Patient views on telemedicine for abortion care and support

Patients discussed the use of telemedicine in relation to all stages of the abortion experience. All but one of the patients interviewed had received telemedical care at some stage of their abortion – most commonly for their consultation and/or aftercare. Overall, patients supported the use of telemedicine and considered it to have played a valuable role at each stage of the patient journey.

### Decision-making

Few patients reported engaging with health professionals during the process of deciding whether to have an abortion. One who did so was disappointed with the telephone support she received. Finding it more ‘clinical’ and less like counselling than she had expected, she felt that in-person support might have been more beneficial for her (ID247, patient, age 36–40 years, home MA, Scotland).

### Referral and access

According to their accounts, patients most often found information online, without speaking to a health professional, about whom to contact to seek an abortion. Patients rarely reported using telemedicine for a referral. A minority reported contacting their general practitioner by telephone as a first step to identifying an abortion provider.

Consultation was the stage of the patient journey at which remote care by phone was most commonly used, and patients widely viewed this as helpful and convenient. Perceived benefits of not having to attend a clinic for consultation included savings in time and cost; shorter wait times; and ease of fitting the consultation into their schedule, including not having to take time off work and disclose to employers. Several patients felt more comfortable talking about what they saw as a sensitive topic by phone. Feelings of embarrassment or a sense of being judged by providers were seen as heightened in face-to-face encounters. As one patient described, ‘talking or asking about something that you’re ashamed of really. That's a lot easier than having to sit directly in front of them’ (ID303, patient, age 16–20 years, home MA, Scotland). One patient reported explicitly that their preference was influenced by fear that anti-abortion protestors might be encountered outside of the clinic, while several others referenced protestors as a negative aspect of their experience or reported reservations about being seen to enter a facility whose sole purpose was abortion provision.

Among patients expressing a preference for an in-person consultation, reasons for doing so varied. A few patients thought that in-person contact might be important for patients experiencing abuse, including lack of control over the decision to have an abortion, a finding that contrasted with the suggestion in free-text comments by health professionals that telemedicine could increase access for patients experiencing abuse. Some patients preferred an in-person consultation for the opportunity it presented for more visual explanation of options and procedures; for others, it was the opportunity to ask questions or for their providers to pick up non-verbal cues; and for many it was simply, as one put it, the ‘personal touch’ (ID123, patient, age 21–25 years, home MA, England) that was considered more easily achieved face to face. As one patient described:

…it's just reassuring and to have the other people in the room so they can look at you if they need to do rather than going by the full by-phone examinations or consultations, even though …it might not be medically needed… (ID188, patient, age 26–30, home MA, Scotland)

As another said, ‘…even though they’ve got the pamphlet and they’ve got an online video explaining how to do everything and what's going to happen… there is something nice about someone sitting with you in person and explaining all of that’ (ID103, patient, age 31–35 years, home MA, England).

One suggestion for a means of compensating for the loss of a personal touch was the use of video calls:

…it would have been nice to have been offered the option of a Zoom one instead of it just being on the phone because from a counselling background as well I think that having that eye contact with someone and I think for me I would probably felt a little bit calmer about that and a little bit more in the moment with it… (ID266, patient, age 36–40, home MA, England)

A recurrent theme in patient accounts was the need for flexibility and provision of options. Phone consultations, it was noted, may work well in conjunction with in-person appointments at other stages of the patient journey, and when combined with other supporting modes of communication, for example, information provided in videos or sent via email or post:

The woman that I spoke to, when she was on the phone she said, ‘The leaflet is through email’, so it was in front of her [the provider], so I could read it but she was also reading it out with us…. So I had plenty of information and I always feel like it's nicer to talk to someone about it rather than sitting and reading through stuff. I feel like I take it in more when someone's talking through with us. (ID203, patient, age 26–30, home MA, England)

Giving patients choices was seen as important, enabling them to select the type of consultation that suited them best, for example:

I think even if they gave it as option. You know, would you like to come in or we can do a telephone consultation? If somebody didn’t feel safe in their own home they could say well I’ll just come in, rather than sit on their phone for an hour. Having it as an option would probably be pretty good. (ID265, patient, age 31–35 years, surgical abortion, Scotland)

### Managing the abortion procedure

Most patients found managing an MA at home with telemedical support unproblematic, but some concerns did emerge at this stage of the process. These related mainly to whether the support would be available when needed and how promptly it could be accessed. A tension emerged in patients’ accounts between the benefits of self-management and the need for reassurance and support. In the absence of the physical presence of a health professional, emotional support from other sources emerged as an important element of home management. One patient who was able to find a friend to provide in-person support was aware that this could be a gap in the self-managed model for patients unable to source support from their own network:

I think maybe the one downside I think was happening…obviously you need somebody there to observe you. And I think as I say, for me, because I live alone, and I don’t really have any family, and because the father wasn’t involved. I asked a friend who was luckily able to come, but it would be good if there was some sort of service where they could provide someone or put you in touch with somebody who is a volunteer who is happy to sit and wait. (ID162, patient, age 31–35 years, home MA, Scotland)

Specific concerns during the procedure were often rooted in a mismatch between expectations and actual experience of pain or bleeding, and patient worry over whether what they experienced was ‘normal’. The absence of a health professional itself could cause some uncertainty and concern even where there were no medical complications:

It was quite scary doing stuff like this at home because obviously there's not any professional, like the professional people, so you don’t know if you’re doing it right or something could go wrong and stuff like that, but it was okay. I think I did it all right. (ID295, patient, age 16–20 years, home MA, Wales)

Additionally, some felt the need for stronger pain relief than that provided in their packs, which was unavailable for home-managed MAs.

Patients appreciated having a telephone number to call directly during the process of a home-managed MA, especially an all-hours service:

….they said, ‘you can call these phone numbers they will be aware that who you are and you’ll be doing it on that day so you won’t have to worry about interrupting or having to explain the situation’…That was very reassuring and nice to know. (ID188, patient, age 26–30, home MA, Scotland)

Where patients had medical concerns, even a short delay when seeking telephone support could cause stress, as a participant who worried about the heaviness of her bleeding described:

So I suppose if I was in a clinic…they would have been able to tell me straightaway. So the 10 minutes that it took for them to answer the helpline was quite scary for the both of us [the participant and her boyfriend]. (ID138, patient, age 21–25, home MA, England)

It transpired, however, that not all patients used the service. For example, one reported not calling despite ‘bleeding quite badly’ (ID244, patient, age 16–20 years, home MA, England) because it was late in the evening and she assumed that no one would pick up.

Some thought that in-person care during the procedure could be more reassuring, both in terms of the availability of health professionals to respond to questions or concerns and because if a medical problem did arise they would be in a healthcare facility with staff who could offer immediate help. The importance of choice was repeated at this stage of the patient journey:

[if] the only choice is for it to be online and at home could lead to quite a lot of anxiety in those groups of people. It's very much back down to choice. Being given a choice is the most empowering thing that you can do to someone, especially medically wise because so many choices are taken away from us already… (ID266, patient, age 36–40, home MA, England)

Patients recommended additional resources to help address these limitations of self-managed abortion, such as written accounts of others’ experiences that would demonstrate the spectrum of what is considered medically normal or answers to commonly asked questions available through the provider website, for example,

…if you just have maybe something about the discharge that comes out, or does this look right, you know a bit of a bot on Google or on the BPAS website, or whatever clinic you are with or ‘I have had an experience of a headache, is that normal?’ (ID118, patient, age 26–30, home MA, England)

### Aftercare and follow-up

Many patients saw telemedical support following their abortion as beneficial. Phone services were widely used where there were problems or concerns post-abortion or a need for counselling. Patients made suggestions for additional applications, including reminders via automated message, email or telephone of the timing of the pregnancy test to confirm completion. Some patients expressed a need for a more general check-in post-abortion and an opportunity to debrief about their experience, share any problems experienced, raise any ongoing concerns and arrange contraception. Most who did felt these needs could be met via telemedicine. They felt that beyond providing a number to call, which patients did tend to find reassuring, more proactive support would also be helpful – for example, someone contacting them to check in. One patient suggested that a person providing this support might ask some questions and provide reassurance: ‘did it go okay…how you bled and kind of reassure you, that's all fine…maybe asking a bit about how you’re feeling, that’d be nice’ (ID125, patient, age 31–35 years, home MA, England). Another patient recommended a video link for this stage, ‘…to actually physically see the person…sometimes seeing our face is a little bit better than hearing just the voice’ (ID287, patient, age 36–40 years, surgical abortion, England).

Patients whose clinics did call to check in on them felt better supported. Some patients worried that if they needed aftercare via telemedicine, they would not be able to access timely support. Actual experiences, however, were mixed, with some accessing remote aftercare smoothly and others struggling to get through by phone or encountering a long waitlist for services. Counselling for emotional support stood out as an aspect of aftercare for which patients felt in-person support could be important. Online counselling could be seen as ‘generic’ and ‘not very personable’ (ID147, patient, age 21–25 years, home MA, England), while it could be considered ‘…hard to actually gauge how someone is feeling…’ by telephone (ID232, patient, age 21–25 years, home MA, Scotland).

## Discussion

### Summary of findings

Drawing on health professional and patient views from across Britain, our data show strong support for the use of telemedicine in abortion care and suggest how it might be optimised to meet diverse patient needs. Among health professional participants, which included a range of current and potential abortion providers from randomly sampled services, less than a quarter opposed the use of telemedicine in abortion care. Their views mostly concurred with those of patients in seeing the logistical benefits of telemedicine in terms of convenience, comfort and speed. For patients, there were additional advantages in terms of greater privacy, ease of disclosure and freedom from embarrassment, while health professionals recognised advantages for the number of patients who could be treated.

Reservations about the use of a telemedical approach were expressed more rarely. For patients, these centred most significantly on the extent to which remote care provided adequate emotional and psychological support, particularly during management of the procedure and for post-abortion counselling. For health professionals, reservations mainly related to fears about the safety of MA without ultrasound. Both health professionals and patients saw the need for telemedical approaches to be complemented and/or extended at different points in the patient journey by additional sources of information and support, and some saw telephone consultations as working well in conjunction with a subsequent in-person visit. Both highlighted the importance of offering modes of care tailored to individual patient needs and preferences.

### Strengths and limitations

A key strength of this study lies in its design. The combination of quantitative and qualitative data collection methods generated rich data, and the sampling strategy allowed exploration of the perspectives of both patients and health professionals. Researchers have previously looked at both but in separate studies, without comparison.^28,29^ The study also benefitted from including both patients with experience of MA and those with experience of surgical abortion and those from different regions of Britain. Our approach to data collection and analysis allowed investigation of the advantages and disadvantages of use of telemedicine at different points in the patient journey, revealing time-specific considerations.

This study has some limitations. In the patient sample, despite purposive sampling and an adaptive, tailored approach to recruitment, no patient participants disclosed experiencing an abusive relationship, and the number of women under 20 years old was small. Although we aimed to look at continuities and divergencies between patients and practitioners, the data were not strictly comparable: health professionals were not specifically asked about the patient journey, and their views were not made in response to questions systematically asked by researchers. Furthermore, free-text comments may not represent the views of health professionals overall; those with experience of complications consequent on remote care, for example, may have been more likely to provide additional information.^25^

### Contextualisation and interpretation

Perceptions of the benefits of telemedicine in both medical and surgical abortion are confirmed in several studies and include its capacity to afford autonomy, privacy, comfort and convenience to patients. The number of patients in our sample having surgical procedures was too small to compare pre-operative consultations in-person and via telemedicine, but quantitative studies have shown no difference in satisfaction between the two.^30^ Areas in which telemedical approaches were seen as needing to be strengthened are confirmed in fewer studies. Despite general affirmation of telemedical approaches among health professionals and in national and international clinical practice guidelines,^1,13,31^ heath professionals’ concerns about the possible risks of incorrect determination of gestational age without routine ultrasound have been observed by others^29^ and need to be taken seriously. Research shows a preference among patients for not having an ultrasound,^28^ adverse outcomes of a fully telemedical model without ultrasound have been shown to be rare,^3,32^ and clinical guidelines state that routine ultrasound scanning is not required as part of abortion care.^13,31,33^ A national cohort study of 52,142 patients accessing early medical abortion in England from January to March 2020 examined the prevalence of cases in which gestational age was reported after the abortion to be greater than the anticipated 10 weeks.^3^ Among the 29,984 patients who did not receive a pre-treatment ultrasound, this occurred in 11 cases, or 0.04%, none of which resulted in additional medical complications.^3^ The need to incorporate such evidence into health professional education and training is underlined by the evidence from our study of greater acceptability of telemedicine among experienced abortion providers and those confident of their skills in supporting home management.

From the patients’ perspective, the finding that some felt more comfortable with a consultation without in-person contact aligns with the findings of other studies.^28,34^ So too does the contrary preference expressed by some for in-person care.^10,34^ For these patients, the absence of opportunities for detecting facial cues, observing body language and making eye contact during the consultation, and for some form of visual supervision during the procedure, were limitations.

We identified a need for emotional and psychological support in addition to medical information and advice during telemedically supported abortion among some participants. Widely documented,^11,28,35–40^ this is an important and thus far underdeveloped component of abortion quality of care.^41^ Again, it may be that for some patients, this can only be addressed by replacing telemedicine with in-person care or by combining the two. Sub-optimal uptake of remote personal support during home-managed MA in our study has been observed by others.^9,11^ Effective signalling of the reliable availability of such a service is clearly important. This finding aligns with Hoggart et al.'s^36^^, p6^ research finding that reassurance of an ongoing connection with and support from the provider can foster ‘a sense of feeling cared for’ among patients. Familiarity with the full spectrum of normal bleeding and discomfort could also help lessen discomfort and anxiety. As patients suggested, this might be achieved by incorporating answers to commonly asked questions into the websites of abortion services and making other patients’ stories accessible to those undergoing a home-managed MA. Confidential online support, for example, which is offered by specialist agencies such as Abortion Talk's ‘Talkline’, can offer opportunities for patients to reflect on their experience of abortion.^42^ Other studies suggest that this would be welcomed by patients and health professionals alike.^32,43^

### Implications for policy and practice

The NICE guidance on abortion care recommends healthcare professional training for those who may encounter patients requesting abortion should include the opportunity for experience in abortion services.^13^ Our findings suggest that training should also include evidence on new models of care, including on telemedically supported MA where no ultrasound is indicated, so that healthcare professionals have a thorough understanding of the associated risks and benefits in context and are equipped to provide accurate information.

The views of health professionals and patients suggest a range of possible options for maximising benefits and addressing perceived shortcomings of telemedically supported abortion care. For a minority of patients, in-person support might better suit their needs. Compared with other groups, higher proportions of patients under age 20 and those identifying as Black, African, Caribbean or Black British have been shown to prefer face-to-face care.^10^ Our data suggest that in-person care might also be important in some cases where there is doubt about whether the patient is making an autonomous decision free of undue influence by others. These findings support national guidelines that in-person care should remain an option for those who prefer it.^1^

For other patients, options exist for complementing telemedical support with alternative models of care. Hybrid approaches include combining a telemedical consultation, for example, with in-person care at other stages of the patient journey. As patients suggest, telemedical consultations can be complemented by provision of further information via digital modes of communication (e.g. video and email) as well as by post. For the procedure itself, where telemedicine is used to support self-managed MA, comprehensive information about the spectrum of normal bleeding and discomfort and about the evidence base supporting the safety of home-managed MA would benefit patients. Furthermore, patients self-managing an MA would benefit from the availability of, and reassurance about, readily accessible remote support for the duration of their abortion procedure and for aftercare. Our findings also suggest that telemedical post-abortion counselling could be limited in its ability to meet patients’ emotional support needs, highlighting the importance of offering in-person counselling at this stage.

There is also scope across the patient journey for the adoption of innovative telemedical approaches to further enhance the abortion experience. The use of video platforms in consultations, where feasible, would provide the opportunities for exchanging non-verbal communication and help to compensate for the lack of visual cues. Incorporating supplemental information for patients into abortion service websites, including about other patients’ experiences, could help patients understand the full range of pain and bleeding that can be expected during a home-managed MA. Telemedicine also offers novel avenues for post-abortion care, including reminders of the timing of the pregnancy test to confirm abortion completion. While capacity limitations may present challenges to in-person follow-up support, automated methods (via, e.g. an SMS, email or telephone) could feasibly provide the aftercare requested by some patients.^25^

Our study confirms the importance of choice to patients, and the need to offer care tailored to their different needs.^25^ It also underlines the need to seek the views of health professionals and patients on optimal models of care.

## Conclusions

Support for the incorporation of telemedicine in abortion provision across stages of the patient journey is high, and this mode of delivery offers a range of benefits in terms of the service, logistics and patient comfort. Service providers should allow patients to choose the mode(s) of delivery best suited to their needs. Some patients require or prefer in-person care, and this should be offered. Telemedical consultations combined with information provided via other channels can improve the patient experience. Novel ways of providing telemedical support should be explored to enhance remote models of abortion care.

## Supplemental Material

sj-docx-1-dhj-10.1177_20552076241288717 - Supplemental material for Patient and health professional attitudes towards the use of telemedicine for abortion care in Britain: Findings from the SACHA study Supplemental material, sj-docx-1-dhj-10.1177_20552076241288717 for Patient and health professional attitudes towards the use of telemedicine for abortion care in Britain: Findings from the SACHA study by Rebecca Meiksin, Maria Lewandowska, Rachel H Scott, Melissa Palmer, Ona McCarthy, Natasha Salaria, Patricia A Lohr, Jill Shawe, Rebecca Sophia French, Kaye Wellings and in DIGITAL HEALTH

sj-docx-2-dhj-10.1177_20552076241288717 - Supplemental material for Patient and health professional attitudes towards the use of telemedicine for abortion care in Britain: Findings from the SACHA study Supplemental material, sj-docx-2-dhj-10.1177_20552076241288717 for Patient and health professional attitudes towards the use of telemedicine for abortion care in Britain: Findings from the SACHA study by Rebecca Meiksin, Maria Lewandowska, Rachel H Scott, Melissa Palmer, Ona McCarthy, Natasha Salaria, Patricia A Lohr, Jill Shawe, Rebecca Sophia French, Kaye Wellings and in DIGITAL HEALTH

sj-docx-3-dhj-10.1177_20552076241288717 - Supplemental material for Patient and health professional attitudes towards the use of telemedicine for abortion care in Britain: Findings from the SACHA study Supplemental material, sj-docx-3-dhj-10.1177_20552076241288717 for Patient and health professional attitudes towards the use of telemedicine for abortion care in Britain: Findings from the SACHA study by Rebecca Meiksin, Maria Lewandowska, Rachel H Scott, Melissa Palmer, Ona McCarthy, Natasha Salaria, Patricia A Lohr, Jill Shawe, Rebecca Sophia French, Kaye Wellings and in DIGITAL HEALTH
