# Supplementary material for: Patient and health professional attitudes towards the use of telemedicine for abortion care in Britain: Findings from the SACHA study
Source: Digit Health. 2024 Nov 3;10:20552076241288717. doi: 10.1177/20552076241288717 (PMC11536586; doi:10.1177/20552076241288717)
Supplement: sj-docx-1-dhj-10.1177_20552076241288717 - Supplemental material for Patient and health professional attitudes towards the use of telemedicine for abortion care in Britain: Findings from the SACHA study [file sj-docx-1-dhj-10.1177_20552076241288717.docx]

**Appendix A. Characteristics of health professionals surveyed**

| **Characteristics of health professionals** | **N^a^** | **% of sample** |
| --- | --- | --- |
| **Gender** |  |  |
| Female | 669 | 87.6 |
| Male | 93 | 12.2 |
| Non-binary |  | Ƚ |
| **Age group** |  |  |
| Under 30 | 84 | 11 |
| 30-39 | 208 | 27.2 |
| 40-49 | 210 | 27.4 |
| 50 or over | 263 | 34.4 |
| **Years since qualification** |  |  |
| < 5 | 97 | 12.7 |
| 5-10 | 166 | 21.8 |
| 11-20 | 216 | 28.2 |
| >20 | 284 | 37.3 |
| **Country** |  |  |
| England | 560 | 72.7 |
| Wales | 79 | 10.3 |
| Scotland | 131 | 17 |
| **Service type** |  |  |
| Specialist abortion | 247 | 25.7 |
| General practice | 156 | 20.3 |
| Maternity | 198 | 25.7 |
| Pharmacy | 54 | 7.0 |
| SRH clinic | 115 | 14.9 |
| **Profession** |  |  |
| Doctor | 176 | 23 |
| Midwife | 261 | 34.2 |
| Nurse | 266 | 34.7 |
| Pharmacist | 62 | 8.12 |
| **Total** | 771 | 100 |

^a^ Where samples do not add to 771, this is due to missing responses

ƚ Counts of 5 or less
