# Supplementary material for: Patient and health professional attitudes towards the use of telemedicine for abortion care in Britain: Findings from the SACHA study
Source: Digit Health. 2024 Nov 3;10:20552076241288717. doi: 10.1177/20552076241288717 (PMC11536586; doi:10.1177/20552076241288717)
Supplement: sj-docx-2-dhj-10.1177_20552076241288717 - Supplemental material for Patient and health professional attitudes towards the use of telemedicine for abortion care in Britain: Findings from the SACHA study [file sj-docx-2-dhj-10.1177_20552076241288717.docx]

**Appendix B. Characteristics of patients interviewed**

|  | **N** | **%** |
| --- | --- | --- |
| Nation  England  Scotland  Wales | 25  20  3 | 52.1  41.7  6.3 |
| Age group  16-20  21-25  26-30  31-35  36-40  41-45 | 6  12  10  11  8  1 | 12.5  25.0  20.8  22.9  16.7  2.1 |
| Ethnicity  Asian/Asian British  Black/African/Caribbean/Black British  Mixed/Multiple ethnic groups  White  Other ethnic group, or not specified | 3  2  1  38  4 |  |
| Previous child(ren)  Yes  No | 14  34 | 29.2  70.8 |
| Previous abortion  Yes  No  Not reported | 17  30  1 | 35.4  62.5  2.1 |
| Abortion method  Home medication abortion  Home medication and surgical abortion  Hospital medication abortion  Surgical | 37  1  2  8 | 77.1  2.1  4.2  16.7 |
