# Supplementary material for: Patient and health professional attitudes towards the use of telemedicine for abortion care in Britain: Findings from the SACHA study
Source: Digit Health. 2024 Nov 3;10:20552076241288717. doi: 10.1177/20552076241288717 (PMC11536586; doi:10.1177/20552076241288717)
Supplement: sj-docx-3-dhj-10.1177_20552076241288717 - Supplemental material for Patient and health professional attitudes towards the use of telemedicine for abortion care in Britain: Findings from the SACHA study [file sj-docx-3-dhj-10.1177_20552076241288717.docx]

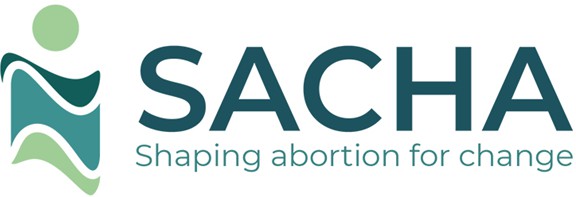

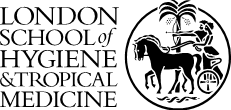

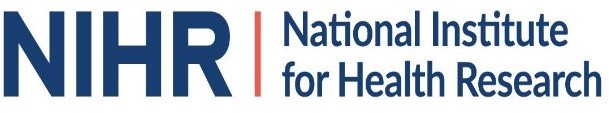


| **2. Do you currently provide any abortion support or care**  **(including pre and post abortion):** | | |
| --- | --- | --- |
|  | **Yes** | **No** |
| On site, i.e.in person with patients |  |  |
| Remotely, e.g. via telemedicine or telephone |  |  |

| **5. Indicate the extent to which the following could prevent those in the same profession as you from taking additional roles in abortion** | | | | |
| --- | --- | --- | --- | --- |
| *NOTE: Those working in abor- tion services will be asked “**Indicate the extent to which the following could prevent other non- abortion specialist healthcare practitioners from taking additional** | **NOT AT ALL** | **TO SOME EXTENT** | **GREATLY** | **DON’T**  **KNOW** |
| Time constraints |  |  |  |  |
| Not enough support staff |  |  |  |  |
| Disapproval of friends/family |  |  |  |  |
| Disapproval of colleagues |  |  |  |  |
| Disapproval of patients |  |  |  |  |
| Harassment from others |  |  |  |  |
| Lack of renumeration for additional role |  |  |  |  |
| Inadequate clinical equipment |  |  |  |  |
| Inadequate digital services (e.g. video) |  |  |  |  |
| Lack of back-up in case of complications |  |  |  |  |
| Inadequate training |  |  |  |  |
| Lack of adequate clinical facilities |  |  |  |  |
| Lack of integrated commissioning |  |  |  |  |
| Difficulties obtaining indemnity cover |  |  |  |  |

| **3. Please indicate if you feel adequately skilled by experience or training to perform the following. Tick all that apply** | |
| --- | --- |
| Helping patient decision-making about pregnancy options |  |
| Counselling on how to take abortion medication |  |
| Informing women what to expect during a medical abortion |  |
| Prescribing abortion medication |  |
| Dispensing or administering abortion medication |  |
| Supporting women in abortion home management |  |
| Discussing disposal of products of conception - home abortion |  |
| Carrying out surgical abortion up to 14 weeks gestation |  |
| Carrying out surgical abortion at 14+ weeks gestation |  |
| Inspection of products of conception to ensure completion |  |
| Providing contraceptive counselling |  |
| Contraceptive implant insertion |  |
| Intrauterine device insertion |  |
| Assessment of post-abortion complications |  |

Please respond by ticking the boxes which correspond with the answer you would like to give. Your answers to these questions will be kept confiden- tial. No attempt will be made to link your professional or personal identity to the data you provide. When you have completed the questionnaire, place in the stamped addressed envelope and post to Natasha Salaria at LSHTM.

**STUDY ID: XXXXXX**

| **1. Please tick whether the following statements in relation to**  **abortion in Britain are true (T) or false (F) or if you don’t know**  **(DK)** | | | |
| --- | --- | --- | --- |
|  | **T** | **F** | **DK** |
| 8 weeks is the upper gestation for women to have a medical abortion at home |  |  |  |
| Women must have an ultrasound before having a medical abortion |  |  |  |
| Having a medical abortion at home is as safe as having a medical abortion in a clinic. |  |  |  |
| Abortion rates are higher amongst those aged 35+ years compared to those under 18 years |  |  |  |
| An abortion is a criminal offence unless it has been signed off by a doctor. |  |  |  |
| Less than 60% of abortions are currently medical abortions. |  |  |  |

| **4. Please enter the number for the responses below that indicates your current involvement in abortion provision:**  **1 = I regularly provide this**  **2 = I provide this sometimes, but would be willing to do more**  **3 = I provide this sometimes, but would NOT be willing to do more**  **4 = None currently, would be willing (with training if needed)**  **5 = None currently, would NOT be willing in the future** | |
| --- | --- |
| Helping patient decision-making about pregnancy options |  |
| Counselling on how to take abortion medication |  |
| Informing women what to expect during a medical abortion |  |
| Prescribing abortion medication |  |
| Dispensing or administering abortion medication |  |
| Supporting women in abortion home management |  |
| Discussing disposal of products of conception - home abortion |  |
| Carrying out surgical abortion up to 14 weeks gestation |  |
| Carrying out surgical abortion at 14 weeks+ gestation |  |
| Inspection of products of conception to ensure completion |  |
| Providing contraceptive counselling |  |
| Contraceptive implant insertion |  |
| Intrauterine device insertion |  |
| Assessment of post-abortion complications |  |

| **6. With training, which other practitioners do you feel should be able to deliver the following aspects of care? *Please tick all that apply*** | | | | | | | | |
| --- | --- | --- | --- | --- | --- | --- | --- | --- |
|  | **GENERAL PRACTITIONERS** | **NURSES IN GENERAL PRACTICE** | **MIDWIVES** | **PHARMACISTS** | **NURSES IN SRH CLINICS** | **DOCTORS IN SRH CLINICS** | **HOSPITAL-BASED 0&G DOCTORS** | **HOSPITAL-BASED 0&G NURSES** |
| Helping patient decision-making about pregnancy options |  |  |  |  |  |  |  |  |
| Counselling on how to take abortion medication |  |  |  |  |  |  |  |  |
| Informing women what to expect during a medical abortion |  |  |  |  |  |  |  |  |
| Prescribing abortion medication |  |  |  |  |  |  |  |  |
| Dispensing or administering abortion medication |  |  |  |  |  |  |  |  |
| Supporting women in abortion home management |  |  |  |  |  |  |  |  |
| Discussing disposal of products of conception - home abortion |  |  |  |  |  |  |  |  |
| Carrying out surgical abortion up to 14 weeks gestation |  |  |  |  |  |  |  |  |
| Carrying out surgical abortion at 14+ weeks gestation |  |  |  |  |  |  |  |  |
| Inspection of products of conception to ensure completion |  |  |  |  |  |  |  |  |
| Providing contraceptive counselling |  |  |  |  |  |  |  |  |
| Contraceptive implant insertion |  |  |  |  |  |  |  |  |
| Intrauterine device insertion |  |  |  |  |  |  |  |  |
| Assessment of post-abortion complications |  |  |  |  |  |  |  |  |

| **7. Indicate your agreement or disagreement with the following statements** | | | |
| --- | --- | --- | --- |
|  | **AGREE** | **NEITHER AGREE NOR DISAGREE** | **DISAGREE** |
| Extending roles in abortion care has the potential to increase job satisfaction |  |  |  |
| Abortion should not be carried out after 12 weeks’  gestation |  |  |  |
| Women should always attend a clinic or hospital to have an abortion. |  |  |  |
| Wider healthcare practitioner involvement in abortion provision ensures a more holistic service for women |  |  |  |
| Abortion at any gestational age is against my personal beliefs |  |  |  |
| More medical abortions at earlier gestations may lead to more women regretting their decision |  |  |  |
| Healthcare practitioners’ objections to providing abortion care negatively affects women’s care |  |  |  |
| Abortion care should be standard practice in my speciality |  |  |  |
| The choice to have an abortion should be completely that of the woman |  |  |  |
| Extending roles in abortion care will be burdensome for health care professionals |  |  |  |
| Digital technologies, e.g. via video, are not an acceptable way to provide abortion care/support |  |  |  |
| Abortion is a health not a legal issue and should be treat- ed as such |  |  |  |
| Healthcare practitioners should counsel women with an unplanned pregnancy on all options available |  |  |  |
| The policy changes enabling both abortion pills to be taken at home should continue regardless of COVID |  |  |  |
| I do not consider that the service I work in should provide abortion support and care. |  |  |  |

| **8. How long ago did you qualify?** |  |
| --- | --- |
| Less than five years ago | 1 |
| 5-10 years ago | 2 |
| 11-20 years ago | 3 |
| Over 20 years ago | 4 |
| **9. Are you:** |  |
| Female | 1 |
| Male | 2 |
| Non-binary | 3 |
| Other | 4 |
| Prefer not to say | 5 |
| **10. What is your current age?** |  |
| Under 30 | 1 |
| 30-39 | 2 |
| 40-49 | 3 |
| 50 or over | 4 |
| **11. How important is religion in your life?** | |
| Very important | 1 |
| Quite important | 2 |
| Not important | 3 |
| Prefer not to say | 4 |
| **12. How would you describe your political beliefs?** | |
| Right/right of centre | 1 |
| Centre | 2 |
| Left/left of centre | 3 |
| None | 4 |
| Prefer not to say | 5 |


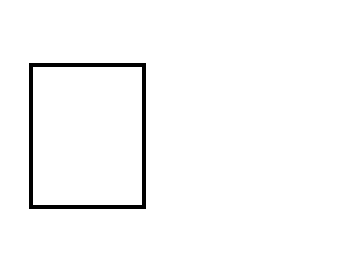

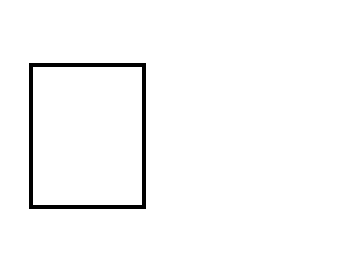

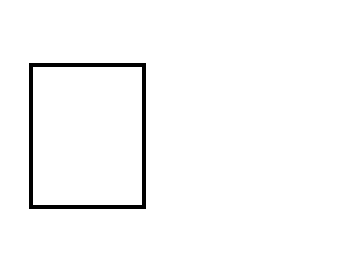

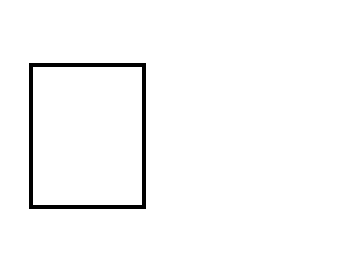

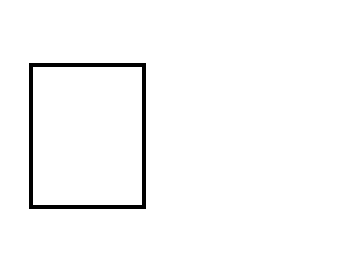

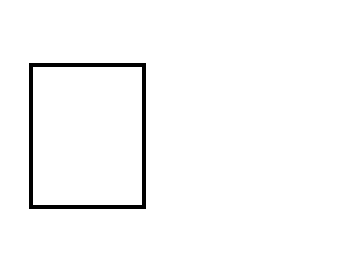

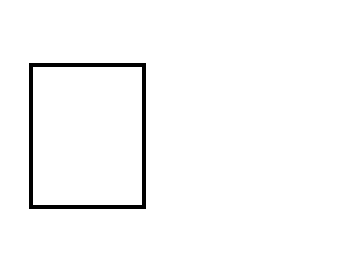

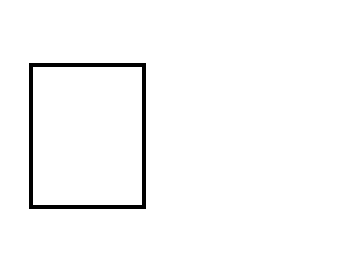

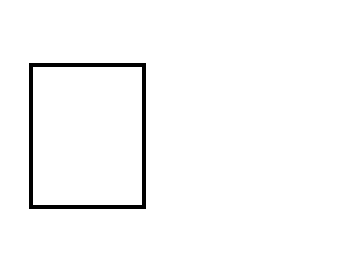

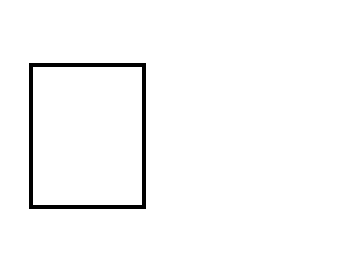

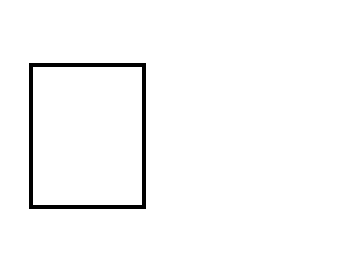

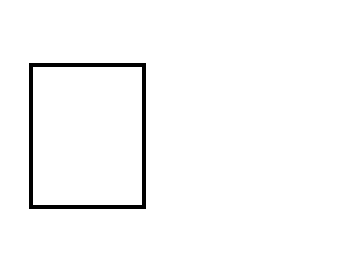

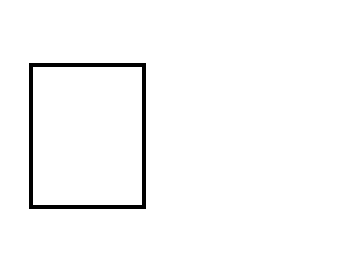

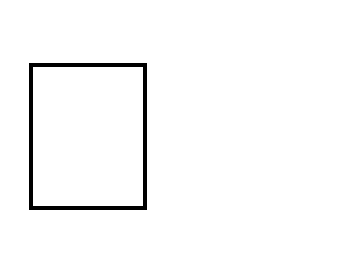

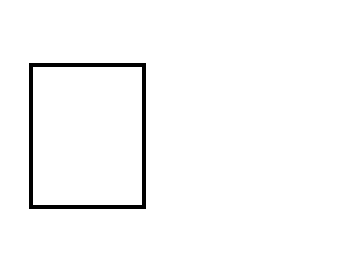

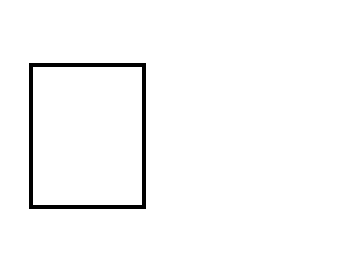

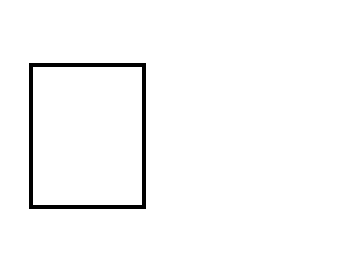

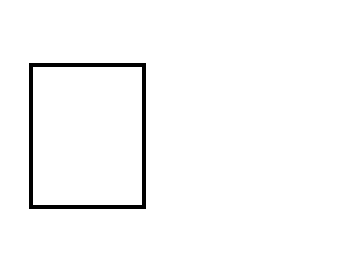

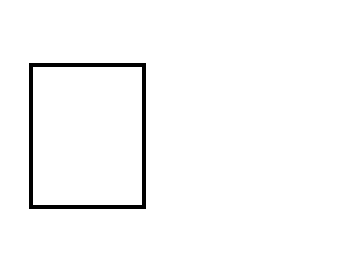

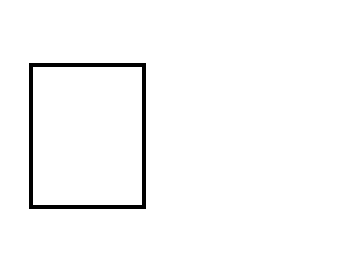

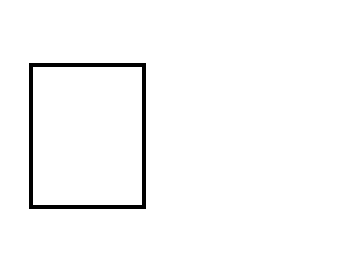

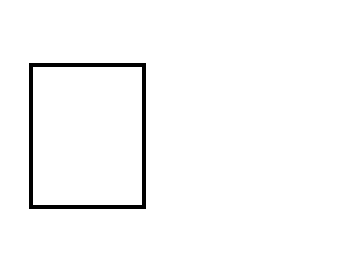


**Please provide any comments:**

**Thank you!**
